# Editorial: Innovation in Glaucoma

**DOI:** 10.3389/fmed.2022.863131

**Published:** 2022-03-03

**Authors:** Michele Lanza, Dariusz Dobrowolski, Soosan Jacob

**Affiliations:** ^1^University of Campania Luigi Vanvitelli, Caserta, Italy; ^2^Dipartimento di Specialità Mediche, Chirurgiche ed Odontoiatriche, Università della Campania Luigi Vanvitelli, Naples, Italy; ^3^Medical University of Silesia, Katowice, Poland; ^4^Dr. Agarwal's Eye Hospital, Chennai, India

**Keywords:** glaucoma, glaucoma surgery, glaucoma diagnosis, glaucoma treatment, intraocular pressure (IOP), standardized automated perimetry, OCT

Glaucoma is one of the main causes of blindness worldwide, among the various forms existing of this disease, the most diffuse is primary open-angle glaucoma but many other types of glaucoma are known. Nowadays, this disease is defined as a multifactorial optic neuropathy characterized by a progressive optic nerve damage causing loss of retinal ganglion cells and their axons, developing specific visual field abnormalities, affecting eyes with open anterior chamber angles.

The goal of its treatment is to conserve the quality of life and the visual function of patients by lowering the intraocular pressure (IOP). Among the possible therapeutic options, physicians can choose a medical therapy with topical glaucoma drugs, laser, or surgical therapy.

Last years have witnessed many innovations both in glaucoma diagnostics and in treatments.

Early diagnosis is always considered a key factor in the successful management of every disease and more, the chronic and subtle ones such as glaucoma. OCT is considered an extremely important device also in glaucoma management. Lehmann et al. suggest considering retinal ganglion cell layer thickness as a very useful tool in highlighting the first alterations in eyes difficult to classify. Tong et al. provided an interesting review showing the relevance of OCT evaluations, both peripapillary and both in macular area to be very helpful in distinguish pre-perimetric glaucoma, early perimetric glaucoma and ocular hypertension.

Another interesting contribution about OCT and Glaucoma came from Chen X. et al. that detected a significant correlation between vessel density measured by OCT angiography and changes in IOP.

Hirooka et al. conducted a very interesting study evaluating morpho-functional changes in glaucoma eyes comparing results obtained with standardized automated perimetry (SAP), electroretinograms (ERG) and OCT showing data that could be very useful both in better understand this disease.

Others helps in improving the timing and the sensitivity of glaucoma diagnosis came from Wen et al., purposing a low contrast visual acuity test to check visual acuity in glaucoma patients, and from Saifee et al., purposing a new software to extract data from SAP report imagines.

Two very interesting reviews, one showing the prevalence of primary angle closure glaucoma by Zhang N. et al., and the other one investigating the trends in treatment approaches by Storgaard et al. have been included in this Research Topic.

The more experimental studies purposed in this section are one related to the correlation between the plasma level of Δ9-tetrahydrocannabinol and the IOP fluctuations by Chen J. et al. and the other one providing a new model of chronic ocular hypertension obtainable in laboratory by Mosaed et al..

Ma et al. suggested an innovative model of measuring ocular biomechanical properties.

About innovation in treatments, Zhu et al., purposed a brief report detailing the results of using a drug supposed to be a neuro-enhancer in glaucoma patients: Scutellarin, whereas Zhang H. et al. provided a study showing their experience in combining Microcatheter-Assisted Trabeculotomy and Deep Sclerectomy and Trabeculectomy in young glaucoma patients.

This sections provided very interesting and useful information to better manage the glaucoma patients.

Altogether this collection of articles emphasizes the importance of measuring the CSF to assess visual function in both basic research and clinical settings. It presents some methods to perform and improve those measures, and considers their interpretation and implications.

## Author Contributions

All authors contributed to manuscript revision, read, and approved the submitted version.

## Conflict of Interest

The authors declare that the research was conducted in the absence of any commercial or financial relationships that could be construed as a potential conflict of interest.

## Publisher's Note

All claims expressed in this article are solely those of the authors and do not necessarily represent those of their affiliated organizations, or those of the publisher, the editors and the reviewers. Any product that may be evaluated in this article, or claim that may be made by its manufacturer, is not guaranteed or endorsed by the publisher.

